# Semantic Interference and Facilitation: Understanding the Integration of Spatial Distance and Conceptual Similarity During Sentence Reading

**DOI:** 10.3389/fpsyg.2018.00718

**Published:** 2018-05-29

**Authors:** Ernesto Guerra, Pia Knoeferle

**Affiliations:** ^1^Center for Advanced Research in Education-CIAE, Universidad de Chile, Santiago, Chile; ^2^Department of German Studies and Linguistics, Humboldt-Universität zu Berlin, Berlin, Germany; ^3^Berlin School of Mind and Brain, Humboldt-Universität zu Berlin, Berlin, Germany; ^4^Einstein Center for Neurosciences Berlin, Berlin, Germany

**Keywords:** eye tracking reading, visual context effects, mental representations, competition, situated language processing

## Abstract

Existing evidence has shown a processing advantage (or facilitation) when representations derived from a non-linguistic context (spatial proximity depicted by gambling cards moving together) match the semantic content of an ensuing sentence. A match, inspired by conceptual metaphors such as ‘similarity is closeness’ would, for instance, involve cards moving closer together and the sentence relates similarity between abstract concepts such as war and battle. However, other studies have reported a disadvantage (or interference) for congruence between the semantic content of a sentence and representations of spatial distance derived from this sort of non-linguistic context. In the present article, we investigate the cognitive mechanisms underlying the interaction between the representations of spatial distance and sentence processing. In two eye-tracking experiments, we tested the predictions of a mechanism that considers the competition, activation, and decay of visually and linguistically derived representations as key aspects in determining the qualitative pattern and time course of that interaction. Critical trials presented two playing cards, each showing a written abstract noun; the cards turned around, obscuring the nouns, and moved either farther apart or closer together. Participants then read a sentence expressing either semantic similarity or difference between these two nouns. When instructed to attend to the *nouns* on the cards (Experiment 1), participants’ total reading times revealed interference between spatial distance (e.g., closeness) and semantic relations (similarity) as soon as the sentence explicitly conveyed similarity. But when instructed to attend to the *cards* (Experiment 2), cards approaching (vs. moving apart) elicited first interference (when similarity was implicit) and then facilitation (when similarity was made explicit) during sentence reading. We discuss these findings in the context of a competition mechanism of interference and facilitation effects.

## Introduction

Recent eye-tracking reading experiments on visually situated comprehension (Guerra and Knoeferle, 2014, 2017) have revealed an arguably more subtle interplay between representations of pictures and online sentence processing than previous studies on written (e.g., Gough, 1965; Knoeferle et al., 2011; comic books: Carroll et al., 1992; newspaper advertisements: Rayner et al., 2001) and spoken language comprehension (e.g., Altmann and Kamide, 1999; but see Cooper, 1974; Snedeker and Trueswell, 2003; Dahan and Tanenhaus, 2005; Huettig and Altmann, 2005; Weber et al., 2006; Duñabeitia et al., 2009). By ‘subtle’ we mean non-referential relations such as between spatial motion (of two playing cards) and semantic similarity (of sentential constituents, e.g., battle and war being similar).

At first glance, two playing cards have nothing much in common with ‘battle and war being similar.’ And yet, congruence (vs. incongruence) between spatial and semantic representations (e.g., spatial closeness vs. distance matching semantic similarity vs. dissimilarity, respectively) rapidly modulated participants’ reading times (of which more below). Underlying this modulation may be conceptual metaphors such as ‘similarity is closeness’ or frequent co-occurrence of similar things in close proximity, both causing representations of spatial proximity to be linked to semantic similarity. Whichever is the underlying cause, these modulations pose challenges for accounts of situated sentence comprehension (Knoeferle and Crocker, 2006, 2007; Altmann and Kamide, 2007; but see Guerra and Knoeferle, 2014, 2017) as they endeavor to accommodate the effects of visually-derived representations of objects (e.g., cards in Guerra and Knoeferle, 2014, 2017). They also enrich the discussion about the extent (and limits) of sensorimotor interaction with language processing (e.g., Zwaan, 2014; Mahon, 2015; Myachykov et al., 2017).

Guerra and Knoeferle (2014) demonstrated in three experiments that the distance between two playing cards (moving either closer to each other or farther apart) modulated participants’ reading times of words in an ensuing sentence expressing similarity or difference between two abstract nouns. In Experiment 1 (Guerra and Knoeferle, 2014), the cards appeared and moved either farther apart or closer together. Once they had reached their end point, they turned, revealing two abstract nouns that then appeared in the sentence. In Experiment 2, the cards on critical trials were blank but participants learned the abstract nouns from Experiment 1 in blocks. In Experiment 3, the methods were identical to Experiment 1 but the cards were blank. Across these experiments, sentences expressing similarity (translated from German: e.g., ‘Battle and war are certainly similar…’), elicited shorter first-pass and regression-path duration/total times for matching (moving closer together) than for mismatching (farther apart) card motion, and vice versa for sentences expressing dissimilarity (e.g., translated from German: ‘Peace and war are certainly different’). These spatial-distance modulations of reading times emerged in sentence regions expressing or implying (dis)similarity (e.g., ‘similar’) and seemed unaffected by whether the cards displayed the nouns (Experiment 1) or not (Experiments 2 and 3). Based on their locus and timing, the authors argued that comprehenders rapidly relate spatial distance to semantic similarity representations via co-indexing (i.e., shared indices). A further specification of co-indexing was, however, missing.

To better specify that mechanism and extend the finding to another domain (social relations), Guerra and Knoeferle (2017) changed the sentences. The initial experiments in Guerra and Knoeferle (2014) had featured noun phrase coordination (‘Battle and war are certainly similar…’) and the cards moved closer or farther apart in coordinated motion. Following current accounts of situated sentence processing, Guerra and Knoeferle (2017, p. 46) argued that comprehenders exploit card parallelism (cards moving in coordination) and noun phrase parallelism (the noun phrases were in a coordinate structure). If this visual-linguistic parallelism in the representations was crucial in co-indexing individual nouns and cards, then the related visual-context effects should disappear when the noun phrases are not coordinated by ‘and.’ The card presentation was identical to that of Experiment 1 in the 2014 study (see above). However, facilitation emerged even without noun coordination and when the sentence content was not about semantic similarity but social relations (e.g., in *Sandra trifft ihre Tante unerkennbar gutgelaunt*, ‘Sandra meets her aunt unmistakably cheerfully…,’ ‘cheerfully’ elicited shorter regression path duration when preceding cards moved closer together compared with farther apart, Guerra and Knoeferle, 2017, Experiment 3).

Variation in sentence structure moreover permitted the authors to examine whether spatial-distance representations could co-index at the valenced adverb even if the card-related nouns occupy different argument positions and the readers had only encountered one of the nouns. In the first experiment, the manner of interaction (i.e., friendly or unfriendly), expected to co-index with spatial distance, appeared after both the sentential subject and object (translated literally from German: ‘Sandra meets her aunt cheerfully/grumpily at …’). In Experiment 2, the manner of interaction appeared after the subject (e.g., ‘Sandra’) but before the object (e.g., ‘her aunt’ in ‘Sandra meets cheerfully her aunt at the health center’). Experiment 3 was identical to Experiment 1 except for the inclusion of a neutral adverb (e.g., ‘unmistakably’) before the socially valenced adverb ‘cheerfully,’ effectively delaying comprehenders’ processing of the valenced adverb.

When a mismatch advantage (in terms of processing time) is observed, such effects are often described as an interference effect. Instead, a match advantage has been described as facilitation. The results from Experiments 1 and 3 in Guerra and Knoeferle (2017) showed for sentences expressing friendly relations (at the post-critical and critical regions) shorter reading times when the cards moved closer together (vs. farther apart), and for sentences expressing unfriendly relations, shorter reading times when the cards moved farther apart (vs. closer together). This pattern replicates the facilitation effects reported by Guerra and Knoeferle (2014). Experiment 2 in Guerra and Knoeferle (2017), by contrast, reported rapid interference effects. First pass times at ‘cheerfully/grumpily’ were shorter for sentences expressing friendly relations when the cards had moved *farther* apart compared to closer together, and shorter for sentences expressing unfriendly relations when the cards had moved *closer* together versus farther apart.

The authors accommodated these contrasting results within the Coordinated Interplay Account (Knoeferle and Crocker, 2006, 2007; see also Knoeferle et al., 2014) by appealing to a competition mechanism and changes in the activation and decay of both visually derived spatial representations and language-based representations. This mechanism predicts that competition between weakly versus strongly active visually-based spatial and semantic representations can cause a reversal from facilitation to interference effects. The appeal to an ‘activation level’ of mental representations is motivated – among others – by research on lexical competition and cooperation between related word representations (see, e.g., Chen and Mirman, 2012). Competition of a target word with strongly activated related word representations (e.g., in dense neighborhoods) can interfere with word identification. By contrast, competition with less strongly activated neighboring word representations interferes little with target word identification. In the experiments by Knoeferle and Guerra, word identification is not at issue. However, the idea of competition has been taken up (much earlier) in the psycholinguistic literature on structural disambiguation (e.g., van Gompel et al., 2000; Vosse and Kempen, 2009) and in computational modeling (McClelland and Rumelhart, 1981). We borrow the idea of a competition mechanism and apply it to representations (of semantic or social relations) from a sentence and consider competition of these with (card) representations of distance. Such competition could interfere with sentence interpretation, slowing reading times for semantically related or matching card-sentence representations. Mismatching card-sentence representations (e.g., cards far apart and nouns/sentences conveying semantic similarity) would be swiftly filtered, speeding reading times.

We test this competition mechanism, to better understand the conditions under which visually-based representations (e.g., of spatial closeness) interfere with (vs. facilitate) the processing of semantic representations (e.g., of similarity between abstract nouns). Related interference effects have been reported in the embodiment literature (Richardson et al., 2003; see also Kaschak et al., 2005; Bergen et al., 2007), but these effects have not yet been explicitly accommodated (but cf. Connell and Lynott, 2012). We present an overview of two new eye-tracking reading experiments and associated predictions followed by the results. In the “Discussion” section we relate our findings to the literature, focusing on commonalities and differences between previously reported results and our own. The discussion foregrounds multimodal interference effects and reconciles existing accounts so as to accommodate further experimental outcomes beyond the present paradigm. We close the discussion with conclusions on the time course of non-linguistic visual context effects during sentence processing.

### The Present Study

We embrace the idea that the level of activation of perceptually and linguistically derived representations (as a function of contextual constraints) is key in determining whether the interaction and competition between related representations yields facilitation or interference (see Guerra and Knoeferle, 2017). In two eye-tracking reading experiments, participants inspected a visual context with two playing cards, each of them featuring a written noun. The card presentation for critical items differed from that used in previous experiments (Guerra and Knoeferle, 2014, 2017). In these previous experiments, the cards had first moved to their end position and then turned, revealing nouns. In the present experiments the cards first revealed nouns, and then turned around and moved either farther apart or closer together. The card context was followed by a written sentence and participants were instructed to read the sentence for comprehension and judge its veracity. Finally, participants saw a picture with two playing cards on all trials and decided whether the cards matched those seen before sentence presentation.

For the card context, we assume that processing semantic relations (between the nouns) will facilitate the processing of spatial distance if semantic relations (e.g., similarity) and card distance (closer) match. Such a match for first noun – then distance presentation order should increase the activation of the associated distance representations (compared to when words are presented only after spatial distance or when no words are on the cards). The logic was that by first showing the nouns, followed by their movements, the semantic similarity of the nouns would boost the activation of spatial distance and that strongly activated distance representations would compete with related semantic (similarity) content of the ensuing sentence, eliciting interference and slowing reading, an effect that might be further enhanced if readers purposely attend to the nouns on the cards.

We hypothesize that if participants construe representations of semantic similarity from the nouns on the cards before the cards have moved closer together (or farther apart), these representations would – via their relation to spatial distance – boost the activation of card distance representations. Boosted spatial representations should interfere with processing congruent sentence meaning which in turn should be reflected in reading times. By contrast, if the presentation of semantically related noun pairs before the cards have moved closer together (or farther apart), does not result in a boost from semantic relations to spatial relations (card distance), we should replicate previously reported facilitation effects: Weakly activated spatial representations should guide participants’ attention to representations of sentence-based semantic similarity, facilitating their processing, but not competing with semantic interpretation.

To better understand the effects of boosting spatial representation by semantic similarity, we varied the emphasis given to the words on the cards: In Experiment 1 of the present article, participants were instructed to pay attention to the words presented on the cards and to read them for comprehension. More specifically, participants were told that these words ‘were important’ and they should ‘read them and try to understand them.’ In Experiment 2, the instructions placed no emphasis on the words. Participants were instructed to inspect the visual context and to remember it (instructions were identical to those in Guerra and Knoeferle, 2014).

Regarding the time course of these effects, re-consider that Guerra and Knoeferle (2014) observed interactions between spatial distance and semantic similarity in both early (e.g., first-pass, Experiments 1 and 3) and later (e.g., total-time, Experiments 1 and 2) measures as soon as semantic relations were explicit (‘similar’ vs. ‘different’ and in their Experiment 2 on the second noun, ‘war’). Based on these prior results, interaction effects could emerge in both early and late measures or only in late measures, and we predict their emergence (at least) as soon as semantic similarity is conveyed by the sentential adjective. If strong competition of representations is in play, we might see delayed effects while weakly active distance representations might prime and elicit rapid facilitative effects in first pass times.

## Experiment 1

We aimed to investigate how the level of activation of perceptual representations (instantiated via the presentation order of nouns and cards) affects the qualitative pattern of interaction between spatial distance and semantic similarity. Thus, we used an existing paradigm and set of materials, shown to result in a facilitation pattern of interaction, to address this issue. By changing the order in which semantic (noun relations) and spatial (card-distance) information is made available in the trial in the present experiment (vs. earlier experiments), and by asking participants to pay attention to the nouns, we expect to boost subsequent processing of spatial distance, which should reverse the influence of congruent spatial representation on semantic processing, resulting in an interference interaction effect.

### Method

#### Participants

Thirty-two native speakers of German with normal or corrected-to-normal vision participated in the experiment for a monetary compensation of 6 Euros each. None of them had been exposed to another language before age six, and all of them read and signed an informed consent form.

#### Material and Design

The images of two playing cards (sized 155× 265 pixels each) were presented as visual context. For all critical trials, each card had a noun written (black font 12 pts.) on the front side. The backside of the cards had a stereotypical blue playing-card pattern. For every trial, both cards appeared at a fixed starting point aligned to each other on the horizontal axis. In critical trials, we manipulated whether the cards moved farther apart or closer together along this axis. We used the 48 critical sentences expressing similarity (or dissimilarity) from a previous study (see Guerra and Knoeferle, 2014 for more details). Each of these sentences had two versions, one expressing similarity between two abstract nouns (‘Battle and war are certainly similar, suggested the anthropologist’) and one expressing dissimilarity between two abstract nouns [‘Peace and war are certainly different, suggested the anthropologist,’ see Example (1)]. Within each item, words that differed were matched for frequency and length.

We combined the two levels of spatial distance (close vs. far) introduced through the visual context, and the two levels of similarity (similar vs. dissimilar) from the written sentences using a 2×2 within-participant Latin square design. Each of the resulting four experimental lists contained each experimental item in one condition. Thus, each participant saw every item only once and the same number of items per condition. In addition to the 48 critical items, each experimental list contained the same 96 filler items. Visual contexts on filler trials resembled experimental trials but varied as to the movement end point of the cards (e.g., upper or lower corners, center of the screen). One third of these trials featured nouns on the cards, while the other two thirds had blank cards. Filler sentences contained a variety of contents (but none about similarity) and structures, some similar to those of the experimental items.

#### Procedure

**FIGURE 1:**
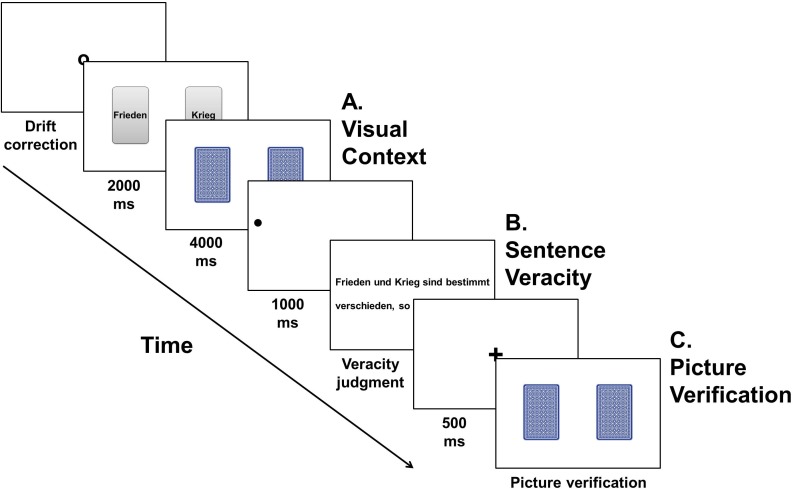
Schematic representation of an experimental trial for Experiment 1.

Participants completed the experiment in a single session. We recorded their eye movements using an Eyelink 1000 desktop head-stabilized tracker (SR Research). A calibration procedure was carried out at the beginning of the session and whenever necessary during the experiment (e.g., after a break). Before starting with the experiment, participants completed 12 practice trials. All trials (including practice trials), were initiated by the experimenter as soon as the participant fixated a central dot, allowing for constant monitoring of the calibration drift. **Figure 1** presents the sequence of events for an experimental trial.

Every trial began with a black dot in the center of the screen for participants to fixate. As soon as the trial was initiated, two playing cards appeared on the computer screen exposing their front side. On all critical trials (and a third of the fillers), the cards showed a written noun each, which participants were instructed to carefully read and understand. After 2 s, cards turned around exposing their blue backside, and only then moved either farther apart or closer together on critical trials. Participants were instructed to pay attention to the visual context and remember it. Four seconds after the cards had turned around, they automatically disappeared from the screen (cards stayed in their final position for about 3.2 s). Subsequently, a fixation dot appeared on the left of the screen and remained for 1 s, to mark the beginning of the sentence. Participants were instructed to fixate the dot until the sentence appeared, then read the sentence carefully, understand it, and judge its veracity against their world knowledge. After participants had made this judgment by pressing a ‘*yes*’ or a ‘*no*’ button (Cedrus Response Pad 8-Buttons, Large), they were presented with two playing cards showing their backside. Participants verified via a button press whether these were identical to the playing cards presented before the sentence.

#### Data Analysis

Raw fixations were filtered before the inferential analysis was carried out. Contiguous fixations shorter than 80 ms duration were merged into a single fixation. Single fixations below 80 ms or longer than 1,200 ms duration were removed (see, e.g., Sturt et al., 2010; Guerra and Knoeferle, 2014). In addition, all incorrect responses were excluded from the analysis. Sentences in the critical conditions were divided into eight different areas of interest, as in (1). Based on previous work (Guerra and Knoeferle, 2014), we defined two areas of interest as critical, namely the NP2 (where similarity is implied by the conjunction of the abstract nouns) and the ADJ (where similarity is made explicit). These regions are marked in bold in (1).

(1) *Frieden*_NP1_ *| und*_coord._ *|****Krieg***_NP2_ *| sind*_VP1_ *| bestimmt*_ADV_ *|* ***verschieden***_ADJ_, *|*

*das verriet*_VP2_ *| der Anthropologe*_NP3_.

‘Peace_NP1_ | and_coord._ | **war**_NP2_ | are_VP1_ | certainly_ADV_ | **different**_ADJ_, |

suggested_VP2_ | the anthropologist_NP3_’

We report three reading measures: first-pass reading times, regression path duration and total reading time per region of interest. First-pass reading times were computed as the sum of all fixations on a region from first entering the region until exiting it. Regression path duration was calculated as the sum of all fixations from first entering a region until moving to the right of that region. This measure includes not only fixations on the critical region but also regressive fixations on previous regions of the sentence. Finally, total reading time is the sum of all fixations in a given region (see, e.g., Liversedge et al., 1998; Rayner, 1998; Traxler et al., 1998; Rayner and Liversedge, 2004). All reading measures were calculated using the Data Viewer software (SR Research). Visual inspection and subsequent one-sample Kolmogorov–Smirnov test of these data revealed that overall reading times were negatively skewed. Consequently, reading times above or below 2 standard deviations of the mean per participant per condition were removed (4.5% of all data) and the remaining data were log transformed before statistical analysis (see Tabachnik and Fidell, 2007). After outlier removal and log-transformation, substantial improvement of the skew was observed for all reading measures and regions (all KS *Zs* < 2, except for regression path duration and first-pass reading times for the second noun region).

To evaluate the effects of spatial distance on reading times we used a linear mixed-effects regression analysis (LMER, lmerTest Package for R). Such statistical models allow the inclusion of crossed random factors for participants and items in a single step as an alternative to separate by-participants by-items analysis (F1, F2 analyses, quasi-F; see Clark, 1973). Participant and item variation around the fixed effects can also be taken into account in the model by including random slopes for participants and items. This is an advantage when analyzing psycholinguistic data, where considerable variance is expected to arise from linguistic materials and participants (see Baayen et al., 2008; Barr et al., 2013). From the LMER analysis estimates, standard errors, *t*-values and *p*-values are obtained for main and interaction fixed effects.

In our mixed model, the two factors and their interaction (i.e., spatial distance and semantic similarity) constituted the fixed effects. Predictors were centered, and participants and items were included as crossed random intercepts. The main effects of the two factors and their interaction were included in the model as random slopes. The final model included fixed effects for the two factors, the interaction between them, participants and items as random intercepts, and both main and interaction effects as random slopes for both random intercepts^1^.

### Results

**FIGURE 2:**
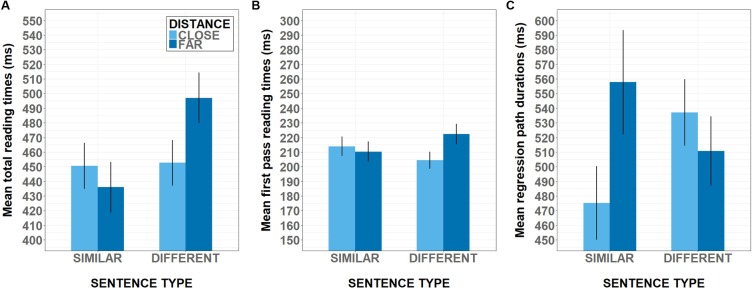
Mean reading times at regions with reliable interaction effects between spatial distance and semantic similarity. The *x*-axis plots the two sentence types (expressing similarity vs. difference) for each level of distance (far vs. close). **(A)** Shows mean total reading times at adjective (ADJ) in Experiment 1. **(B)** Shows first pass reading times at the second noun phrase region (NP2) in Experiment 2. **(C)** Shows regression path duration at the adjective (ADJ) in Experiment 2. Error bars plot the within-subjects adjusted standard error of the mean.

**Table 1** presents mean reading times in milliseconds per condition for all regions of interest in three reading measures and 95% confidence intervals adjusted for within-subject designs (Rouder and Morey, 2005; Cousineau and O’Brien, 2014). The results from the LMER analysis on log-transformed reading times showed at the first critical region (NP2) neither main, nor interaction effects in any reading measures (all *ts* < |2|). By contrast, a reliable interaction effect between spatial distance and semantic similarity was found at the ADJ region in total reading time (2.12, *p* < 0.05). **Figure 2A** shows the pattern of interaction observed in this region in milliseconds. Total reading times were longer for sentences that expressed similarity when preceded by cards closer together (vs. farther apart), while sentences expressing dissimilarity had longer reading times when preceded by cards farther apart (vs. closer together).

**Table 1:** Mean reading times in milliseconds and within-subject adjusted 95% confidence intervals (by condition, region and measure) in Experiment 1.

			First-pass		Regression path		Total time	
					
Region	Condition		Mean	CI 95%	Mean	CI 95%	Mean	CI 95%
**NP2**	SIMILAR	CLOSE	214,39	18.3	244,30	25.2	499,57	54.3
		FAR	213,63	16.7	259,65	27.2	533,95	54.0
	DISSIMILAR	CLOSE	226,31	19.3	268,55	28.5	489,34	51.3
		FAR	214,65	17.2	255,77	23.3	467,33	39.9

**ADJ**	SIMILAR	CLOSE	350,99	20.7	477,52	43.3	450,59	31.1
		FAR	334,17	21.2	476,22	45.6	435,98	34.1
	DISSIMILAR	CLOSE	345,78	19.6	501,70	38.8	452,62	30.6
		FAR	342,07	20.7	491,81	40.6	497,03	34.1


### Discussion

Existing evidence shows that spatial distance facilitated processing of semantically congruent sentences (Guerra and Knoeferle, 2014, Experiment 1). Compared with that study, we modified the moment at which semantic information (via a pair of related nouns) was presented in the visual context and instructed participants explicitly to pay attention to the nouns, keeping the materials and the experimental design the same. With these changes, we observed the opposite pattern of interaction compared with the results by Guerra and Knoeferle (2014, Experiment 1). At the ADJ, we found that total reading times were longer for sentences expressing similarity when cards had moved closer together (vs. farther apart), while for sentences expressing dissimilarity longer total reading times emerged when cards had moved farther apart (vs. closer together). The effects reported here emerged in a relatively late reading measure (i.e., total reading times) compared to previous findings in which spatial distance effects appeared in first-pass reading times at the same sentence region. One potential explanation might be that the emphasis given to the words-on-cards (by presenting the nouns first and instructing participants to pay attention to them) increased the competition of representations and/or as a result perhaps the participants’ cognitive load, resulting in spatial distance modulating sentence reading in later measures (i.e., total reading times) and causing competition, eliciting an interference pattern. Alternatively, perhaps semantic interference occurs only late during language processing (see, e.g., Mahon et al., 2007 for a related discussion in the context of picture-word integration).

## Experiment 2

In Experiment 2, we used identical materials, methods, and in particular card presentation, except for one change: Compared to Experiment 1, no emphasis was given two the words on the cards (i.e., participants were not instructed to read and understand the words on the cards). If the delay in the time course of the observed interference effects arose because participants deliberately focused their attention on the two words (boosting card distance representations and perhaps resulting in strong competition with semantic similarity representations from the sentence), then this delay should decrease, and effects at the ADJ emerge earlier. Alternatively, if interference between congruent semantic and card representations is invariably delayed, interaction effects should emerge again in late (e.g., total time) measures at the ADJ.

Instead of more rapid interference, we might, however, observe facilitation. If in-depth processing of the noun pairs is necessary to elicit interference, then–in the absence of instructed attention to the noun pairs–facilitation effects could emerge: Recall that explicit attention to the nouns may boost the activation of semantic similarity and boost the activation of related spatial-distance representations, yielding strong competition with sentential relations of semantic similarity. In the absence of explicit attention to the nouns per instruction, competition between card distance and semantic similarity representations from the sentence might be relatively weak (since card distance representations would be less active), facilitating (rather than interfering with) the processing of sentential semantic relations.

### Method

#### Participants

Thirty-two further participants took part of the experiment. They were all native speakers of German with normal or corrected-to-normal vision, and had not been exposed to a second language before age six. They read and signed an informed consent form and received a monetary compensation of 6 Euros for their participation.

#### Materials, Design, Procedure, and Data Analysis

The materials, design, procedure and data analysis were identical to Experiment 1, except that unlike in Experiment 1, the instructions did not prompt participants to pay attention to words on the cards. We applied the same outlier threshold as in Experiment 1 (removing 4.2% of total data). Reading times were log-transformed resulting in improved skew values (all KS *Zs* < 2, except for regression path duration for the second noun region).

### Results

**Table 2** presents reading times in milliseconds per region of interest, measure and experimental condition (95% confidence intervals corrected for within-participant designs).

**Table 2:** Mean reading times in milliseconds and within-participant adjusted 95% confidence intervals (by condition, region and measure) in Experiment 2.

			First-pass		Regression path		Total time	
					
Region	Condition		Mean	CI 95%	Mean	CI 95%	Mean	CI 95%
**NP2**	SIMILAR	CLOSE	213,89	13.1	275,91	29.8	544,61	58.3
		FAR	210,23	13.5	309,31	40.0	519,28	54.9
	DISSIMILAR	CLOSE	204,40	11.3	295,45	43.2	528,65	48.1
		FAR	222,28	13.8	290,84	32.9	488,02	47.6

**ADJ**	SIMILAR	CLOSE	317,13	19.5	475,14	49.4	439,33	33.7
		FAR	328,30	18.6	557,78	70.2	462,02	39.3
	DISSIMILAR	CLOSE	331,79	16.9	537,01	44.9	462,31	29.1
		FAR	321,42	18.7	510,74	46.7	432,48	29.3


The LMER analysis on log-transformed reading times shows no reliable main effects but a reliable interaction effect between spatial distance and semantic similarity in first-pass reading times (*t* = 2.09, *p* < 0.05) at the NP2 region. Sentences expressing similarity showed longer reading times when preceded by cards closer together (vs. farther apart), while reading times for sentences expressing dissimilarity were longer when preceded by cards farther apart (vs. closer together). For the ADJ, analyses corroborated a main effect of semantic similarity (*t* = 2.06, *p* < 0.05) with longer regression path times for sentences expressing dissimilarity (vs. similarity). Interestingly, analyses of this measure for the ADJ region also found a significant facilitation effect (*t* = -2.53, *p* < 0.05), replicating the facilitation effect from Experiment 1 in Guerra and Knoeferle (2014), albeit in a later measure (regression path instead of first-pass). Sentences expressing similarity elicited shorter times when preceded by cards closer together (vs. farther apart); sentences expressing dissimilarity elicited shorter times when preceded by cards farther apart (vs. closer together). Total reading time analyses for the ADJ region yielded the same pattern but the effect was only marginally significant (*t* = -1.85, *p* = 0.07). **Figures 2B,C** depict the pattern of interaction in first-pass times at the second noun region and the pattern observed in regression path duration at the adjective region, both in milliseconds. No other main or interaction effects were observed.

### Discussion

In Experiment 1, we showed that with the same materials and a similar experimental setup (Guerra and Knoeferle, 2014), previously reported facilitation effects turn to interference between spatial distance and semantic similarity. We reasoned that presenting the nouns before the cards had moved to their end point and instructing participants to attend to the nouns on the cards, should give an additional activation boost to congruent visual context representations (i.e., distance between cards), interfering with subsequent congruent semantic content instead of facilitating its processing. While we did observe an interference effect, the time course of this effect was delayed relative to previous findings.

In Experiment 2, we asked whether the observed interference effect would emerge in later or earlier measures (e.g., first-pass) if participants were not instructed to focus on processing the noun pairs preceding spatial distance and if such lack of a boost via instructed attention would turn interference into facilitation (replicating Guerra and Knoeferle, 2014, Experiment 1). We kept the changed card presentation (relative to Guerra and Knoeferle, 2014, 2017) such that the cards revealed nouns before moving to their end position. When participants had not been instructed to pay attention to the word pairs, interference effects of card distance emerged in first pass times at the NP2 during sentence reading. Analyses of the data from Experiment 2 revealed another key finding: At the ADJ region, we replicated a previously observed facilitation interaction effect between spatial distance and semantic similarity, albeit in regression path duration instead of first pass times (Guerra and Knoeferle, 2014, Experiment 1). When participants were not instructed to pay attention to the words on the cards, the semantic relations of the nouns may have been processed more superficially. If so, then semantic content (i.e., similarity) would not have had the same boosting effect on the activation of related spatial representation (compared to the present Experiment 1). The interference pattern observed in first-pass at the second noun suggests that the boost of semantic content on spatial distance (during visual context inspection prior to sentence reading), was not completely absent, but as the activation of spatial distance decayed during sentence reading, processing semantic content may have been facilitated for matching (vs. mismatching) card motion.

## General Discussion

**FIGURE 3:**
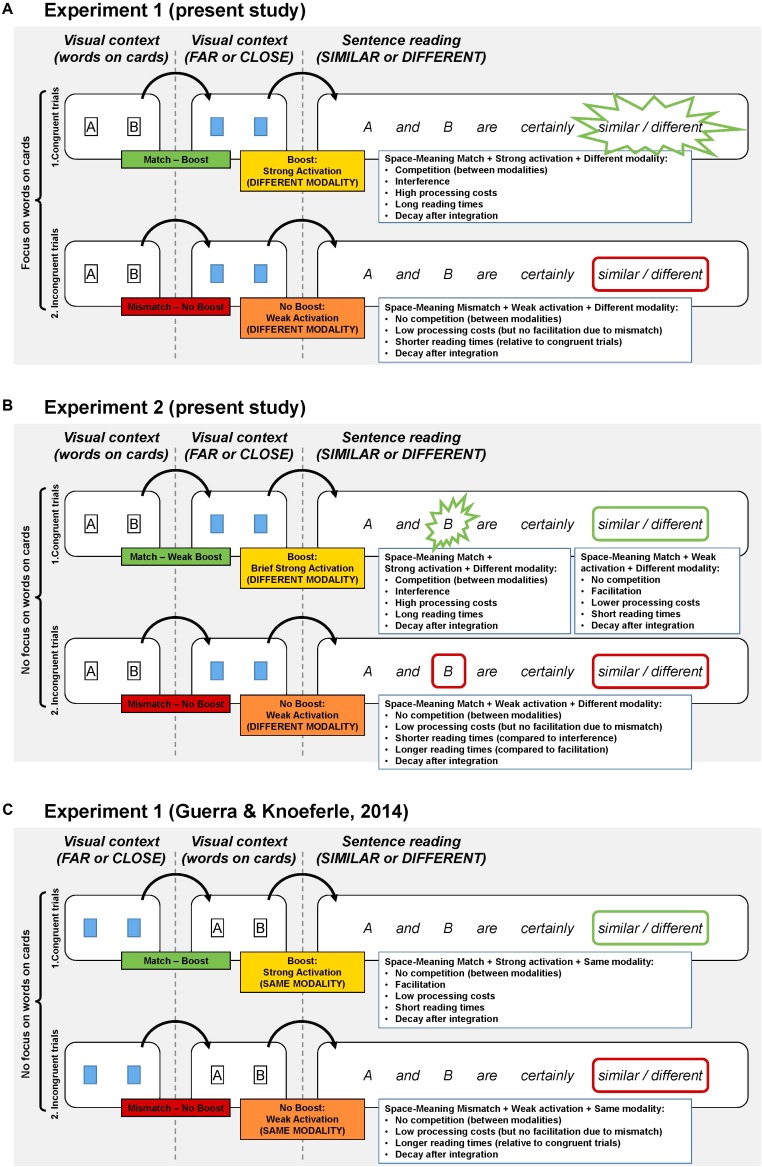
Schematic representation of the hypothesized chain of processes for the present experiments **(A,B)** and Experiment 1 **(C)** from Guerra and Knoeferle (2014). In all three panels, 1 and 2 represent congruent and incongruent trials, respectively. They also show three critical stages: two visual context stages (distance: far or close; words on cards), and a sentence processing stage (conveying semantically similar or different content). Depending on the emphasis given to the words on the cards (A. vs. B. and C) and the presentation order of semantic and spatial information (A. and B. vs. C) the competition account predicts distinct consequences of the interplay between space and meaning on reading times.

In two eye-tracking reading experiments, we assessed whether facilitation effects can be turned into interference when changing only the card presentation and instructions compared with Guerra and Knoeferle (2014). In Guerra and Knoeferle (2014, 2017), two playing cards had first moved farther apart or closer together and then revealed two nouns (**Figure 3C**). This sequence preceded the comprehension of sentences about semantic (dis)similarity. By contrast, in the present experiments, cards showed nouns before moving to their final position (see **Figures 3A,B**). We reasoned that the earlier presentation of the nouns (e.g., battle and war conveying similarity) would contribute to the strength of activation of related spatial-distance representations (e.g., of closeness). More active spatial distance representations might then compete with semantic similarity representations during sentence reading, eliciting interference effects. In Experiment 1, in addition to the changes in card presentation, participants were instructed to pay attention to the nouns and likely processed these in-depth for their semantic relation (similarity vs. dissimilarity); the semantic relation may have boosted the related spatial representations (of closeness vs. distance). We predicted that strongly activated visually derived representations should interfere with, and weakly active visually derived representations should facilitate, the processing of related semantic representations. The idea that competition can slow processing (in ambiguity resolution) receives support from the psycholinguistic literature on constraint-based models (e.g., Traxler et al., 1998; Vosse and Kempen, 2009, see Arbib and Caplan, 1979; McClelland and Rumelhart, 1981 for related computational predecessors).

As predicted, we observed an interference pattern at the ADJ region in Experiment 1. Yet, this effect emerged in a late measure (total time) compared to previous facilitation effects (Experiment 1 in Guerra and Knoeferle, 2014), for which we entertained two possibilities. A first possibility was that by prompting participants to pay special attention to the words-on-cards at the beginning of the trial, we extenuated participants’ cognitive resources delaying semantic processing. Note that in Experiment 1, we only observed a reliable interaction effect and no main effect of similarity; the latter effect had been pervasive in previous studies. Alternatively, it was possible that interference effects only occur in later reading measures. No previous studies have examined the detailed time course of interference effects, thus this remained an empirical question (but cf. Mahon et al., 2007). Previous evidence (cf. Guerra and Knoeferle, 2014, Experiment 2) suggested that the former potential explanation was plausible.

Indeed, in Experiment 2 when participants were not explicitly instructed to pay attention to the words on the cards, we observed an immediate interference effect (i.e., in first-pass reading times at NP2). By contrast, a facilitative interaction pattern appeared in regression path times at the ADJ region. This first-interference-then-facilitation pattern might have emerged per a boosting effect of the two nouns on the cards on spatial distance representations, yet this boost was short-lived compared to Experiment 1. This evidence shows that interference and facilitation can occur within a single experiment, at different time points during semantic interpretation at the sentence level.

### Accounts of Related Interference Versus Facilitation Effects

Not unrelated to the present findings, Richardson et al. (2003) reported that congruence between the spatial relation implied by sentences (e.g., *The balloon floats through the cloud*) and spatial information (e.g., a square presented at the top of the screen) caused interference in a visual discrimination task (Experiment 1) but facilitation in a memory task with the same materials (Experiment 2). While task differences (visual discrimination vs. memory; among other differences in the procedures) could have caused these contrasting results, facilitation and interference effects between object representations and sentence comprehension have also been reported using similar tasks (see Connell, 2007 and Zwaan and Pecher, 2012, Experiments 3a and 3b, for conflicting effects in a replication study).

Richardson et al. (2003) and Bergen et al. (2007) reported interference effects in experiments with non-overlapping, sequential (language-before-picture) presentation of non-integrable stimuli (i.e., a spoken phrase preceded a square/circle at the top or the bottom of the screen). To date, qualitative changes in the interaction between object representations and language processing have been accommodated by appealing to the role of temporal overlap in visually based and language-based information (sequential vs. simultaneous), so-called ‘integrability’ (of which more below) and attentional modulation. A first account (Kaschak et al., 2005) suggests that interference effects should appear when both language and non-linguistic visual perceptual information are presented simultaneously but are non-integrable. The authors’ concept of integrability relies, in our view, on whether the linguistic content directly refers to depicted objects. For instance, “the sentence and the [visual] stimulus are integratible […] when one sees the image of a car while hearing, ‘The car approached you.”’ (Kaschak et al., 2005, p. B86). By contrast, seeing an animation of a black-and-white spiral, creating an illusion of forward or backward movement, is not referentially integratible with “The car approached you,” presumably because no concrete objects can be referenced in the visual depiction. Recall the reported facilitation (Guerra and Knoeferle, 2014) of non-referential context (i.e., spatial distance between cards) on sentence reading times. To the extent that this sort of non-referential relation is non-integrable, the reported *facilitation* effects are inconsistent with accounts that predict interference for non-integrable perceptual/conceptual representations (see Kaschak et al., 2005, p. B86).

In a follow-up study, Kaschak et al. (2006) presented written sentences about object motion (e.g., a rocket starting) together with auditory (not visual) stimuli (such as white noise conveying upward motion, Experiment 1). Cross-modal interference emerged when participants judged the sensibility of the written motion sentence (e.g., *with a deafening sound, the rocket blasted off*), while listening to white noise conveying upward motion (slower responses to matches than mismatches). Kaschak et al. (2006) argued that interference arises when “the auditory perception system is required to process two stimuli involving motion in the same direction (i.e., the sound of the motion described in the sentence, and the sound of the auditory motion stimulus)” (p. 738). Their prediction derives from evidence on cross-modal perception, viz. that for an attentionally demanding task, same-modality task-secondary stimuli are filtered (Lavie, 2005). While simulation seemed impeded in cross-modal presentation, facilitation effects (faster responses to matches than mismatches) emerged within the same experiment in Kaschak et al. (2006) when the sentences were presented in the *auditory* modality together with the white noise.

To accommodate the findings by Kaschak et al. (2006) and Connell and Lynott (2012) propose that “auditory attention was […] occupied in monitoring motion in a particular direction, and so there were insufficient attentional resources free when the sentence called for auditory simulation of motion in the same direction” (p. 2). The logic underlying this reasoning is that sentence reading elicits auditory ‘simulation.’ By ‘simulation,’ Connell and Lynott mean: “representations of objects and events that are not in the current environment […] are functionally comprised of partial replays (i.e., simulations) of the neural activation captured during perceptual, motor, affective, and other experience” (p. 1). They assume that the auditory sentence (that must be judged for sensibility) will have attentional priority. Participants will perceive the white noise but it won’t occupy attention since it is secondary to the sentence-sensibility task. As a result, the white noise directs attention without occupying it, facilitating simulation of same-direction motion for sentence processing in the auditory modality. An account based on attentional modulation (e.g., Connell and Lynott, 2012) would assume that visually-derived spatial information could (in principle) facilitate processing of congruent sentences (perhaps by pre-activating spatial and semantic representations), as long as it is processed prior to sentence comprehension. In this sense, it seems compatible with the results of Guerra and Knoeferle (2014). Connell and Lynott (2012) argued that different tasks might guide modality-specific processing demands and attention in different ways (see also Kreysa and Knoeferle, 2011 on the effects of task). However, their account neither predicts the timing of these effects during comprehension (it is not a processing account) nor how differences in (instructed) attention allocation would modulate visual context influences during real-time sentence comprehension (but see Taylor and Zwaan, 2008).

### Reconciling the Accounts

Semantic facilitation and interference effects have been observed for a variety of experimental tasks and processing levels, such as picture naming (e.g., Mahon et al., 2007), word recognition (e.g., Mirman and Magnuson, 2008), sentence processing (Kaschak et al., 2005; Wassenburg and Zwaan, 2010), and visual discrimination (Richardson et al., 2003; Bergen et al., 2007). But what are the cognitive mechanisms implicated in these effects? Connell and Lynott (2012, p. 1) claim that “[t]he perceptual and attentional systems are intertwined, and, since the conceptual and perceptual systems share modality-specific neural substrates, it should come as no surprise that they also share associated attentional mechanisms” (see also Pecher et al., 2003). This assumption led the authors to give more importance to the role of the attentional modulation as a mediator for the interaction between visual and linguistic representations, instead of temporal overlap or integrability. In our understanding, this account assumes that both facilitation and interference can occur with a simultaneous or sequential presentation of visual-context and sentence stimuli, and both when stimuli are apparently integrable and when they are not integrable. According to Connell and Lynott (2012), interference effects appear if perceptual stimuli occupy modality-shared attentional resources needed to process similar linguistic information. Facilitation, instead, would be observed when stimuli initially direct attention to the perceptual modality and soon after that the focus of attention moves from the perceptually derived representation to the linguistic representation, resulting in priming of related relative to unrelated concepts. In this sense, their account relies on an attentional mechanism.

Undoubtedly, attention allocation plays a major role in cognitive processing. To complete the task in our study, participants had to shift their attention from information conveyed in the visual context (i.e., card distance) to semantic content during sentence reading, while keeping the visually derived non-linguistic representation of spatial distance active in working memory (**Figure 3**). Since this was true in both of our experiments, effects of the post-sentence verification task unlikely caused the contrastive between-experiment results. However, we could appeal to the consequences of attentional demands (per the instructions) on the level of activation of mental representations. When participants purposely focused their attention on the abstract nouns on the cards (Experiment 1), semantic processing may have pre-activated related spatial representations, boosting their level of activation in working memory. By contrast, when participants processed these nouns more superficially (Experiment 2), the level of activation of the spatial distance representation in memory was arguably weaker. These differences in activation strength may have caused the distinct reading-time patterns for the subsequent sentence. Against this background, we think that a mechanism based on competition changes (depending on the level of activation of representations) can accommodate the reviewed results on interference- and facilitation effects.

### Task, Presentation Order, and Integrability

In the present article, we focused on cognitive processes and representations. Different tasks might result in different dynamics of perceptual, conceptual and attentional processing. However, evidence shows that different tasks might elicit the very same processing dynamics. For this reason, concentrating on the task or aspects of the paradigm (such as timing of presentation alone), could be potentially misleading. Let’s re-consider the timing of presentation and integrability. It has been argued that simultaneous presentation of related (but non-integrable) perceptual and conceptual information should result in a mismatch advantage, since “neural mechanisms [necessary for conceptual processing] are already engaged by the visual percept” (Kaschak et al., 2005: p. B86). Non-linguistic visually derived representations may be active while participants are exposed to a visual pattern of moving bars; however, visual-context representations may also remain active beyond the presentation time of the corresponding stimuli (see, e.g., Spivey and Geng, 2001; Altmann, 2004). The results from the present two experiments suggest that sequential presentation of non-linguistic and linguistic information can also result in a mismatch advantage (i.e., interference). Consequently, presentation order does not seem to be decisive in predicting the effect pattern.

With regard to the integrability of informational sources, Kaschak et al.’s (2005, p. B87) account predicts that non-integrable perceptual and conceptual information can only interfere with each other if presented simultaneously, while no effects should be observed when presented sequentially. We argue, instead, that integrability of contextual non-linguistic and language-based information influences the activation level of visually and linguistically derived representations. Tentatively, as integrability increases, the processing time needed for shifts of attention from visually to linguistically derived representations decreases. To the extent that this logic is correct, then non-integrable visually and linguistically derived representations might be more demanding for the cognitive system when informational priorities (e.g., from non-linguistic to linguistic) change. As a result, integrability is not what ultimately determines whether this transition results in interference or facilitation, since it has a constant effect on congruent and incongruent non-linguistic visual context and linguistic information. To the best of our knowledge, no studies have directly compared the influence of integrable and non-integrable representations; therefore, this remains an empirical question. However, the results of our second experiment suggest that both interference and facilitation effects can be observed even when integrability is constant.

In summary, while factors such as timing and integrability might influence the dynamics of the interaction between visual (e.g., card distance) and language-based (e.g., semantic similarity) representations, they do not on their own determine the direction of the effects. Instead, we argue that these factors can contribute to the level of activation of non-linguistic visual context and linguistically derived representations at a given moment, but that it is competition resulting from the activation of mental representations which ultimately elicits facilitation or interference.

### Representations and Time Course

Some comments on the potential overlap of representations from non-linguistic visual and linguistic information appear necessary. Kaschak et al. (2005) seem to assume that non-linguistic visually derived representations are a constitutive part of semantic representations, and that these are related. Similarly, Connell and Lynott (2012), assumed that conceptual processing interacts with the perceptual system. Indeed, decades of eye-tracking evidence have revealed that the language system is highly sensitive to a variety of contextual informational sources, from overt referential relations between nouns and objects to subtler mappings between spatial representation and abstract semantics (see, e.g., Knoeferle and Guerra, 2016 for a review). Yet, we believe that existing evidence has not yet shown unequivocally that perceptual representations have a functional role in conceptual processing (but see Amsel et al., 2014), and recent results suggest that causal relation might be task-dependent (e.g., Ostarek and Huettig, 2017).

What continuous measures such as eye-tracking can do is to deliver detailed insight into the time course with which contextual information modulates language processing. In this sense, our results suggest that both facilitation and interference can emerge in early reading measures as participants read a sentence. They also show, in coherence with previous findings that (just as for referential, lexical-semantic, and thematic role relation mappings) the influence of very subtle perceptual representations on language comprehension is time-locked to the processing of critical sentence regions. In particular, both facilitation and interference effects emerge when related semantic content is implied or explicitly mentioned in the sentence, underscoring predictions of the Coordinated Interplay Account (Knoeferle and Crocker, 2006, 2007) but also of the linguistic focus hypothesis (Taylor and Zwaan, 2008). What our results add is insight into the competition implicated in reconciling visually-derived spatial representations with semantic processing during sentence comprehension.

## Conclusion

In two eye-tracking experiments we intended to further understand conflicting patterns of effects reported in the literature: visually-derived (card distance) information appears to sometimes facilitate related language processing (of sentential semantic relations), and sometimes interfere with it. Existing accounts are either ill equipped to accommodate findings from the literature or too underspecified to make clear processing predictions. We tested a mechanism that appeals to competition and to the activation of visually and linguistically based representations as modulators of facilitation- and interference effects. Our results suggest that weakly active card distance representations facilitate related semantic processing while strongly activated distance representations interfere with it. These findings contribute to a better specification of the mechanisms implicated in the interplay between visually-derived non-linguistic representations and language processing.

## Ethics Statement

At the time we conducted the present studies, the University of Bielefeld did not yet have an official Ethics Committee. Previous inquiries made by members of our research group at the time obtained as a response that if we followed standard procedures for psycholinguistics research obtaining direct ethical approval from a Review Board was not necessary. Note that the all reported research in the present paper was conducted exclusively with adult participants and using experimental methods that pose to the best of our knowledge no risk of physical harm. Moreover, under no circumstance we expose participants to language or visual materials that could threaten their moral integrity. Participants were invited to take part of our study, and before the experiment or any other assessment was conducted participants read an Information Form, in which they could read about the task they would be performing, the absence of known risks related to the methodology in use and about the confidentiality of the data. After that, they had the opportunity to ask questions and then signed an Inform Consent form. This form explicitly stated that the participants could abandoned the experiment at any time if they wished to do so. All participants completed this procedure invariably.

## Author Contributions

EG and PK contributed to the design of the experiments and wrote the manuscript. EG conducted the experiments and analyzed the data.

## Conflict of Interest Statement

The authors declare that the research was conducted in the absence of any commercial or financial relationships that could be construed as a potential conflict of interest.

## Footnotes

1 R-code example: lmer (first-pass ∼ distance ^∗^ similarity + (1 | participant) + (0 + distance | participant) + (0 + similarity | participant) + (0 + distance : similarity | participant) + (0 + distance | item) + (0 + similarity | item) + (0 + distance : similarity | item) + (1 | item), data).
