# Corrigendum: Semantic Interference and Facilitation: Understanding the Integration of Spatial Distance and Conceptual Similarity During Sentence Reading

**DOI:** 10.3389/fpsyg.2018.01417

**Published:** 2018-08-06

**Authors:** Ernesto Guerra, Pia Knoeferle

**Affiliations:** ^1^Center for Advanced Research in Education-CIAE, Universidad de Chile, Santiago, Chile; ^2^Department of German Studies and Linguistics, Humboldt-Universität zu Berlin, Berlin, Germany; ^3^Berlin School of Mind and Brain, Humboldt-Universität zu Berlin, Berlin, Germany; ^4^Einstein Center for Neurosciences Berlin, Berlin, Germany

**Keywords:** eye tracking reading, visual context effects, mental representations, competition, situated language processing

In the published article, Pia Knoeferle was not included as a corresponding author. This has now been rectified. The authors apologize for this error and state that this does not change the scientific conclusions of the article in any way.

The original article has been updated.

## Conflict of interest statement

The authors declare that the research was conducted in the absence of any commercial or financial relationships that could be construed as a potential conflict of interest.

